# In-Liquid Lateral Force Microscopy of Micropatterned Surfaces in a Fatty Acid Solution under Boundary Lubrication

**DOI:** 10.1038/s41598-019-51687-8

**Published:** 2019-10-23

**Authors:** Masaki Tsuchiko, Saiko Aoki

**Affiliations:** Department of Chemical Science and Engineering, School of Materials and Chemical Technology, Tokyo Institute of Technology, 2-12-1 Ookayama, Meguro-ku, Tokyo, 152-8552 Japan

**Keywords:** Mechanical engineering, Surface chemistry

## Abstract

This study aims to investigate the influence of surface morphology on boundary-lubricated friction in a stearic acid solution. The surface morphology was controlled by fabricating submicrometer line-and-space patterns on Si(100) surface via photolithography. The boundary-lubricated friction on the patterns was measured by in-liquid lateral force microscopy for both transverse and longitudinal ridges, with respect to the sliding direction; the highest friction was observed on longitudinal ridges and grooves, which is in agreement with the tendency observed in our previous friction studies on steel surfaces. To further investigate this phenomenon, some additional patterns having different submicrometer morphologies were prepared and their friction characteristics were investigated. On the patterns not allowing the fluid to flow along the grooves, the frictional forces were equivalent for transverse and longitudinal grooves and ridges. Therefore, the high friction observed on the longitudinal ridges was caused by flowing out of fluid along the grooves, and it was possible to conclude that the fluidity around the submicrometer ridges and grooves influences the friction-reducing effect of stearic acid in boundary lubrication regime.

## Introduction

In boundary lubrication, organic polar compounds such as fatty acids form molecular films adsorbed on surfaces, with excellent friction-reducing effects. The Bowden–Tabor model can explain the friction mechanisms of these adsorbed molecular films^1^. In this model, the key factors of frictional forces in boundary lubrication are the shear strengths of solids and tribofilms and the coverage of the molecular films; in other words, in Bowden–Tabor model, the surface morphology and the lubricant used as the fluid have little influence on the boundary-lubricated friction. Therefore, surface morphology has been considered as a factor unrelated to friction in the boundary lubrication regime.

However, recent studies showed that, even under boundary lubrication, surface morphology influences the friction characteristics^2–9^; they were focused on the relation between the hardness of textured surfaces and friction coefficient^2^, influence of dimensions on friction coefficient^6,7^, correlations between roughness parameters and friction coefficient^4^, and dependence of the friction coefficient of textured surfaces on the lubricant molecular structure^3^. These works have pointed out that the surface morphology is an important factor of boundary-lubricated friction but the theory explaining its role in this phenomenon had not been defined yet.

In our previous researches, we further demonstrated the dependency of the friction coefficient, in boundary lubrication regime, on the surface morphology when fatty acid-based oils are used as lubricants^10–13^. When analyzing steel specimens having a grinded surface, we observed lower friction for the grinding direction transverse to sliding compared to the longitudinal one; furthermore, from the viewpoint of the transverse roughness, a correlation between roughness parameters, especially the arithmetic mean peak curvature (S_pc_) and friction coefficient, was observed^13^. These experimental results suggested that the anisotropy of the surface morphology can affect a tribofilm formation or the friction-reducing effects of organic polar compounds. In the case of transverse roughness, a microscopic hydrodynamic pressure arises around asperities due to trapped oil and enhances the formation or orientation of adsorbed molecular films, resulting in a lower friction coefficient. However, this mechanism is only in supposition and the detailed mechanism is still unknown.

Since this mechanism describes the phenomenon at the protrusion scale, we assumed that the frictional force measurements with a microscale contact area as large as the protrusions could be effective for its elucidation. In other words, the reduction of the contact area to the protrusion scale allows the direct comparison of the protrusion shape measured and frictional force without relying on statistics such as roughness parameters for the protrusion shape evaluation. Lateral force microscopy (LFM) has been widely used for experiments with such microscale and nanoscale contact areas^14–23^; among the previous studies, the friction measurement on microsized and nanosized texture surfaces have attracted significant attention because such texture scale was considered as an effective method to control frictional forces in microelectromechanical and nanoelectromechanical systems (MEMS and NEMS), where liquid lubricants cannot be used. Therefore, in these previous researches, frictional force measurements by LFM were performed under dry or very thin film lubrication conditions. On the contrary, this study was focused on in-liquid LFM with an aim to elucidate the mechanism of surface morphology influence on friction characteristics under boundary lubrication. In addition, we used electron-beam (EB) lithography for micropatterning on Si(100) surfaces to improve the controllability in the surface shape fabrication.

Herein, we investigated the relation between the friction characteristics of fatty acid-based oils and anisotropy of surface morphology. The main purpose was to clarify how such friction characteristics change depending on the directionality of anisotropic surface morphology. In particular, we fabricated some patterns on a Si(100) surface via EB lithography, measured the frictional forces by in-liquid LFM with a microscale contact area equivalent to the pattern size, and confirmed the relation between surface morphology and frictional force.

## Materials and Methods

### Patterned surface preparation

**Figure 1:**
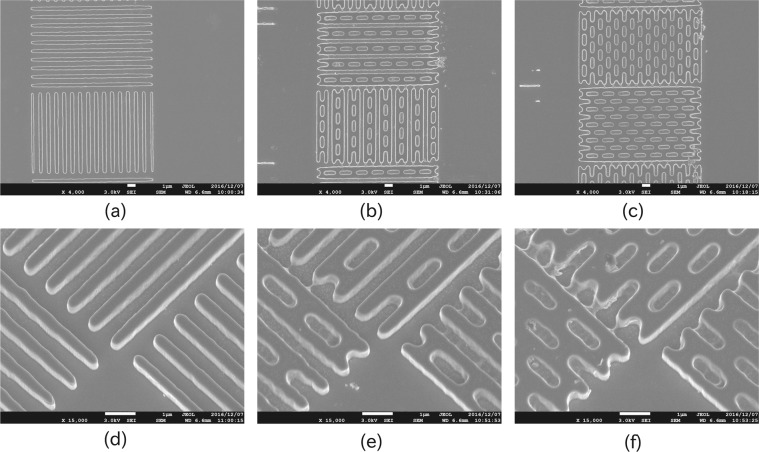
Top (top row) and bird’s eye (bottom row) view scanning electron microscopy images of three different micropatterns, fabricated via EB lithography: (**a,d**) line-and-space (L&S), (**b,e**) L&S + Baffle1, and (**c,f**) L&S + Baffle2 patterns.

To freely control the surface morphology with high precision, we realized the patterns via EB lithography. A Si(100) chip was used as the substrate. We prepared three different patterns, which were observed by scanning electron microscopy with a JSM-7500F microscope (JEOL Ltd.) and whose secondary electron images are shown in Fig. 1. The first pattern, a simple line and space type (L&S pattern), was fabricated to investigate the variation of the frictional force with the direction of the ridge, i.e., the convex portion of the L&S pattern, while the concave portion is defined as groove; both its ridge and groove widths were 500 nm, resulting in a pitch of 1000 nm. As shown in Fig. 1(a), we designed the L&S pattern so that the sets of ridges having different directions were close to each other, which permitted us to include different ridge direction parts into a single LFM image.

In addition to the L&S pattern, two other patterns having baffles in the grooves were fabricated. These patterns were defined as L&S + Baffle1 (L&S + B1) (Fig. 1(b,e)) and L&S + Baffle2 (L&S + B2) (Fig. 1(c,f)), which differed in terms of the baffle density. The width of baffle was 500 nm and a pitch of a baffle was 2000 nm, resulting in 1500 nm of groove length.

The groove depth measured by scanning electron microscopy was 400 ± 10 nm. This error was mainly caused at quantifying process of depth from secondary electron images and we confirmed that the depth of each groove was identical. Since the three patterns were etched with the same RIE batch, they had the same groove depth.

### LFM probe preparation

**Figure 2:**
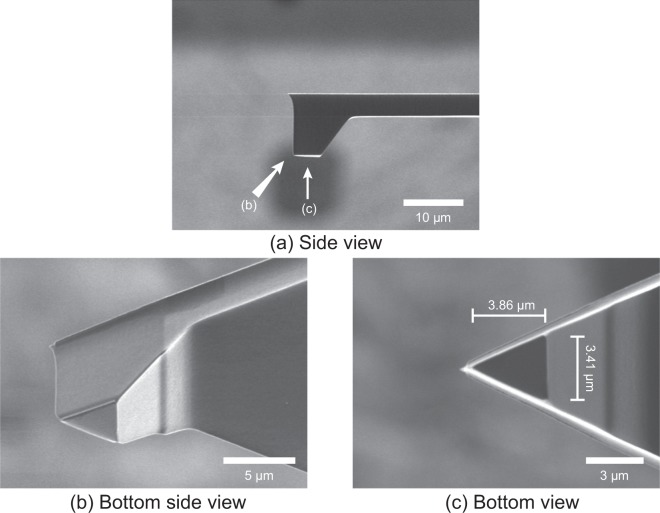
Scanning ion microscopy images of the plateau probe tip obtained with a focused ion beam system and used for the lateral force microscopy measurements.

Since we aimed to investigate the influence of surface morphology on boundary friction, a probe tip larger than the pattern pitch was required; therefore, the LFM measurements were performed with a flattened probe (plateau probe). Otherwise, the LFM measurement would have become a frictional force measurement in a very limited area inside one ridge. The probe was obtained by cutting off the tip of a commercially available, sharp probe (OMCL-AC160TS, Olympus Corporation) and flattened with a focused ion beam system (JEM-9310FIB, JEOL Ltd.); the resulting plateau was triangle shape whose bottom length was 3.41 μm, height was 3.86 μm and area was 6.64 μm^2^, as shown in Fig. 2. To avoid load concentration on the edge of plateau plane, the edge was rounded by focused ion beam.

### LFM measurements

LFM is a mode of atomic force microscopy (AFM), a technique consisting in scanning a probe perpendicularly to a cantilever beam and measuring the frictional force as a twist of the cantilever. Since we were interested in the relation between boundary-lubricated friction and surface morphology, the LFM measurements had to be conducted in lubricated conditions (in-liquid LFM). In this study, two liquid media were used for the LFM measurements; additive free *n*-hexadecane and stearic acid solution (5 mmol/kg). The frictional force on the patterning surface was measured using the plateau probe and a general-purpose AFM unit (SPM-9700, Shimadzu Corporation).

The frictional force measurements were conducted so that both pattern regions, i.e., where the ridge and sliding directions were orthogonal or parallel, were included in one field of view (Supplementary Fig. S1) because, otherwise, it would have been difficult converting the measured values into units of force, hindering the comparison of frictional forces among multiple images. To acquire a single LFM image, a fast scan was performed perpendicularly to the cantilever beam and reciprocated once and a friction loop was obtained at one line; then, with moving the scan position, friction loops at different scan lines were obtained sequentially. We conducted experiments so that the upper and lower areas of the LFM images showed the regions where the ridge and fast scan directions were parallel (longitudinal ridge area) and transverse (transverse ridge area), respectively. The vertical direction of LFM measurements was top-to-bottom direction for all measurements.

The normal load was calculated based on the normal sensitivity derived from the slope of the force–distance curve (FDC) on the Si surface^24^ and the normal spring constant calculated via the general Sader method for arbitrary shape cantilevers^25^. The experimental conditions are listed in Supplementary Table S1. The measurement was following procedure; Firstly, FDC was measured in *n*-hexadecane, followed by the LFM measurements on the L&S, L&S + B1, and L&S + B2 patterns; after the replacement of pure *n*-hexadecane with the stearic acid solution without changing cantilever, the FDC was measured again, followed by other LFM measurements on the three patterns. In this measurements condition, the influence of the drag force by liquid on the lateral deflection was two orders of magnitude smaller than frictional force, hence the effect of drag force was negligible.

### Data processing

During the LFM measurements, one line was reciprocally scanned in the direction perpendicular to the cantilever beam and one image was acquired by sequentially moving this measurement line. Since the frictional forces were measured reciprocally on one line, the friction loop was acquired. The right and left scanning directions of the image were named as trace and retrace, respectively, as shown in Supplementary Fig. S1.

In the LFM measurements, the cantilever was twisted by frictional force and this twist was measured as the lateral deflection of the laser spot, which was reflected on the back of the cantilever and focused onto the photodetector. This lateral deflection was not a force unit but a value without a physical meaning and, in the apparatus used, it was output as voltage in the of ±10 V range. There is a linear relation between lateral deflection and frictional force and its proportional coefficient depends on the lateral spring constant of the cantilever and the lateral light sensitivity^26^. Therefore, when comparing the magnitudes of frictional force by lateral deflections without their conversion into force units, it was limited to comparison between conditions that proportional coefficient did not change, i.e., comparison with the same cantilever and same atmosphere; in other words, at least within the same image, the magnitude of the frictional force can be compared with that of the lateral deflection without its conversion into force units. It should be noted that direct comparisons between two or more images by lateral deflection without calibration into force unit have no physical meanings and should not be made.

The trace-minus-retrace (TMR) value of lateral deflection, which is proportional to frictional force^18^, was calculated for plotting maps and data analysis. In this study, the lateral deflection was not converted into force units. Our main concern was to investigate the frictional behavior changing by the direction of ridges. Therefore, the investigation by not the frictional force in force unit but the differences of friction between transverse and longitudinal ridge region satisfied our requirement. Also, despite the several methods for obtaining coefficients to convert the lateral deflection into force units^27–33^, a conversion coefficient could not be obtained with high accuracy for the plateau probe used and in liquid medium. Because of these reasons, the calibration of the lateral deflection in voltage unit into frictional force in force unit was not carried out.

To evaluate the frictional force magnitude in one image, a statistical analysis was applied. The statistical analysis was carried out as follows. A lateral deflection of 150 × 100 pixels (8.8 μm × 5.9 μm) was extracted from the longitudinal and transverse ridge areas in one LFM image and a histogram was plotted to compare the distribution of the lateral deflections.

To clarify the difference in lateral deflection between longitudinal and transverse ridge areas, the Cohen’s *d*, an effect size, was calculated as follows^34^:

1 $$\begin{array}{rcl}d & = & \frac{\overline{{x}_{L}}-\overline{{x}_{T}}}{{s}_{C}},\\ {s}_{C} & = & \sqrt{\frac{{n}_{L}{s}_{L}^{2}+{n}_{T}{s}_{T}^{2}}{{n}_{T}+{n}_{L}}},\end{array}$$

where $\bar{x}$ is the mean value, *s* is the standard deviation, *n* is the data size (in this case, 150 × 100 = 15,000), and the subscripts L and T indicate the area used for calculating these statistic values (longitudinal and transverse ridge area, respectively). Cohen’s *d* expresses the deviation between the average values of two samples; *d* = 0 means that the averages are the same, whereas large *d* values indicate a great difference. Herein, since the *d* value in this paper was calculated by above-defined equation, the *d* value has sign and a positive value means the average at longitudinal ridge area is higher than transverse one. For the statistical processing and the mapping of the LFM results, we used Python 3.7.

## Results

### L&S pattern

Supplementary Fig. S2 shows topography image acquired via LFM at a normal load of 63.8 μN in pure *n*-hexadecane. The upper and lower left parts correspond to the longitudinal and transverse ridge regions, respectively. No topographic contrast was observed in both cases, confirming that the plateau probe tip was completely on the pattern ridge.

**Figure 3:**
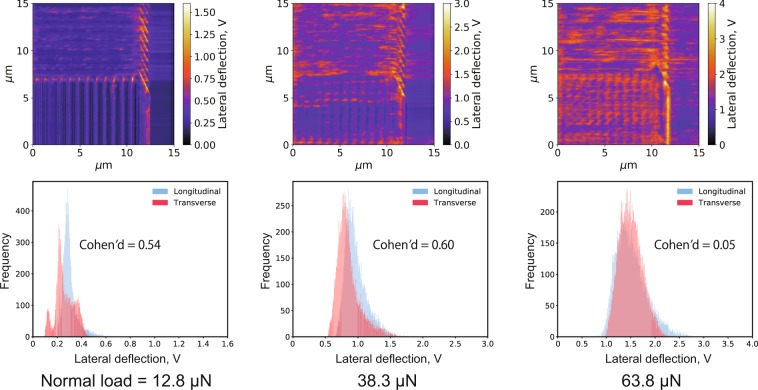
Lateral deflection images (top) and corresponding histograms (bottom) measured at different normal loads in *n*-hexadecane; samples of histograms were extracted from two areas (150 pixels × 100 pixels each) having different asperity direction, transverse and longitudinal, against sliding. Cohen’s *d* is also displayed on histograms.

The lateral deflection images measured in *n*-hexadecane and their histograms extracted from the longitudinal and transverse ridge regions are displayed in Fig. 3, showing that the lateral deflection increased with the load. At the highest load (63.8 μN), high-friction portions appeared in both region; hence, the frictional force did not depend on the ridge direction.

**Figure 4:**
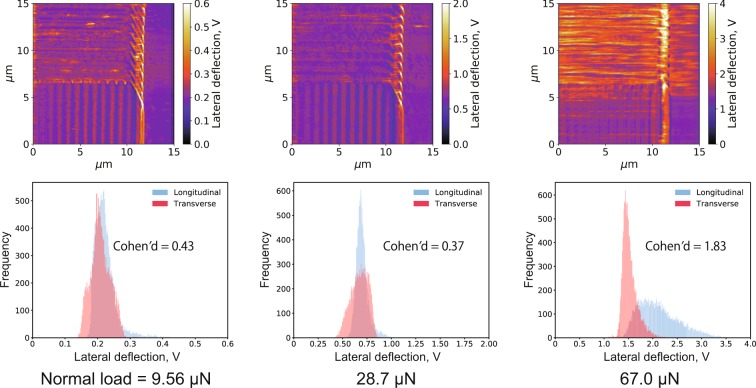
Lateral deflection images (top) and corresponding histograms (bottom) measured at different normal loads in stearic acid solution; samples of histograms were extracted from two areas (150 pixels × 100 pixels each) having different asperity direction, transverse and longitudinal, against sliding. Cohen’s *d* is also displayed on histograms.

Figure 4 shows the lateral deflection images and corresponding histograms measured in the stearic acid solution. These LFM results cannot be compared with those reported in Fig. 3 because they were obtained with different liquid compositions, leading to different proportionality coefficients of lateral deflection and frictional force: therefore, the discussion of their absolute lateral deflection values is not important. At a load of 67.0 μN, a difference in frictional force due to the ridge direction was observed, i.e., it was higher in the longitudinal ridge region than that in the transverse. On the contrary, at lower loads, no clear difference in frictional force arose between the ridge directions.

The Cohen’s *d* values calculated from these results are also displayed in Figs. 3 and 4; the largest value was obtained from the LFM results measured in stearic acid solution under the highest load. On the other hand, for the results in *n*-hexadecane, the *d* values were low, i.e., there was no clear difference in lateral deflection with the ridge direction.

In summary, in the stearic acid solution, lower frictional forces were observed when the ridge and scanning directions were orthogonal to each other compared to the parallel case. Since this tendency was the most remarkable at the highest loads, the following section is focused only on the results measured at such load conditions.

### L&S + B1 and L&S + B2 patterns

**Figure 5:**
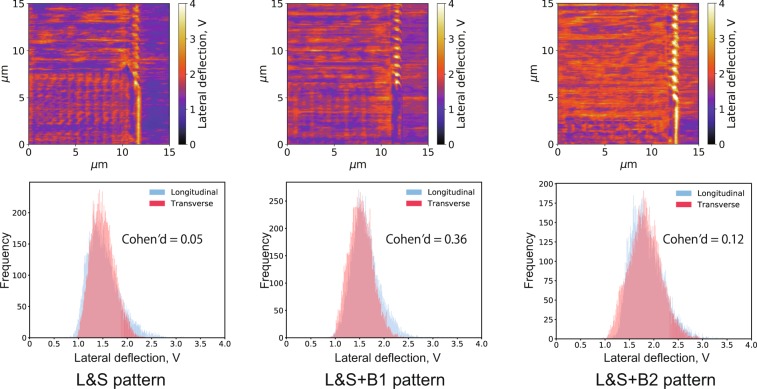
Lateral deflection images and corresponding histograms for the three patterns immersed in *n*-hexadecane at a normal load of 63.8 μN.

Figure 5 shows the lateral deflection images and histograms for both L&S + B1 and L&S + B2 patterns measured in *n*-hexadecane with a normal load of 63.8 μN and compares them with the results for the L&S pattern (also shown in Fig. 3). In all cases, when immersed in *n*-hexadecane, the site of the high frictional force did not depend on the pattern direction.

**Figure 6:**
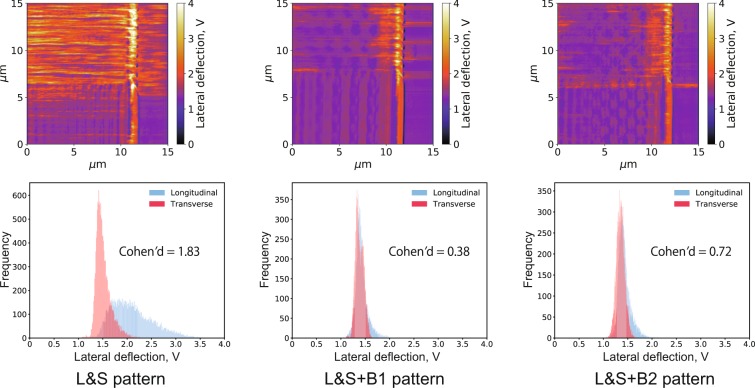
Lateral deflection images and corresponding histograms for the three patterns immersed in stearic acid solution at a normal load of 67.0 μN.

Figure 6 shows the corresponding results at a normal load of 67.0 μN in stearic acid solution. Although the highest lateral deflection was observed at the longitudinal ridge region of the L&S pattern, the lateral deflections slightly differed between transverse and longitudinal ridge regions on the L&S + B1 and L&S + B2 patterns.

The Cohen’s *d* values for the three patterns immersed in *n*-hexadecane and stearic acid solution are also showed in Figs 5 and 6. The highest value (1.83) was observed with the L&S pattern in stearic acid solution, resulting in a larger frictional force in the longitudinal ridges compared with the transverse ones. On the contrary, the L&S + B1 and L&S + B2 patterns immersed in the same solution exhibited remarkably smaller *d* values (0.38 and 0.72, respectively) and the frictional force was no longer affected by the ridge direction. When immersed in pure *n*-hexadecane, all patterns showed small *d* values, clearly demonstrating the independence of the frictional force from the ridge direction.

## Discussion

In Figs 3 and 4, some contrast was observed in lateral deflection image on the unpatterned flat area. This reason is not clear at this moment; however, the small amount wear of pattern, at the extent the pattern structure was not broken, was observed by scanning electron microscopy after all LFM measurements (Supplementary Fig. S3). It may be considered that these wear particles were deposited on the flat area and this caused the contrast on the un-patterned flat region.

In the results measured in *n*-hexadecane shown in Fig. 3, no significant difference in frictional force due to ridge direction was observed even in high normal load condition. Therefore, the ridge and groove directions have no influence on the friction in the *n*-hexadecane environment. On the other hand, based on Fig. 4, in the stearic acid solution, higher frictional forces were measured on the longitudinal ridge region compared to the transverse one especially in high normal load condition, which well agrees with the results of our previous friction tests on steel specimens^13^. Although the contact area scale was greatly different between the LFM measurements (μm) and steel friction tests (mm), the results were consistent between them and the phenomenon reported for the steel friction tests was observed in these in-liquid LFM experiments. Therefore, LFM could be regarded as an idealized system of steel friction testing and applied for investigating the friction anisotropy caused by anisotropic surface morphology.

As shown in Fig. 6, when immersed in the stearic acid solution, the L&S + B1 and L&S + B2 patterns did not exhibit a significant difference between transverse and longitudinal ridge regions. Therefore, it is considered that the higher friction observed in the longitudinal ridge region of the L&S pattern was suppressed by including baffles in the grooves. Some differences due to the pattern shape or direction were thought and, as one of these, the contact area can be thought. The contact area calculated for each pattern and direction is summarized in Table 1 and also the distribution maps of contact area are showed in Supplementary Figs. S4–S6. For calculating the contact area, the plateau dimension (bottom = 3.41 μm, height = 3.86 μm) and pattern dimensions were used. From Table 1, the standard deviation of contact area shows a large difference between transverse and longitudinal direction, in other words, fluctuation of the contact area is larger in the longitudinal region than the transverse region. If it is hypothesized that a contact area strongly affects a lateral deflection, the distribution of lateral deflection must be broader in the longitudinal ridge region compared with the transverse ridge region for all patterns. However, from Figs 5 and 6, the distribution width of lateral deflection was equivalent between the longitudinal and transverse area in both *n*-hexadecane and stearic acid solution for almost all patterns, except the L&S pattern immersed in stearic acid solution. Therefore, it is considered that the contact area has small effects on the frictional force in the apparatus used in this study and the contact area could not be a main reason why Cohen’s *d* shows dramatically large value on L&S pattern in stearic acid solution environment.

**Table 1:** Calculated contact area of each patterns and directions, μm^2^. (Maps of contact area are showed in Supplementary Figs. S4–S6).

	L&S		L&S + B1		L&S + B2	
	Transverse	Longitudinal	Transverse	Longitudinal	Transverse	Longitudinal
Average	3.30	3.30	3.71	3.71	4.12	4.12
Standard deviation	0.065	0.254	0.084	0.281	0.088	0.195
Max	3.39	3.74	3.88	4.49	4.34	4.55
Min	3.21	2.89	3.52	3.14	3.90	3.76

**Figure 7:**
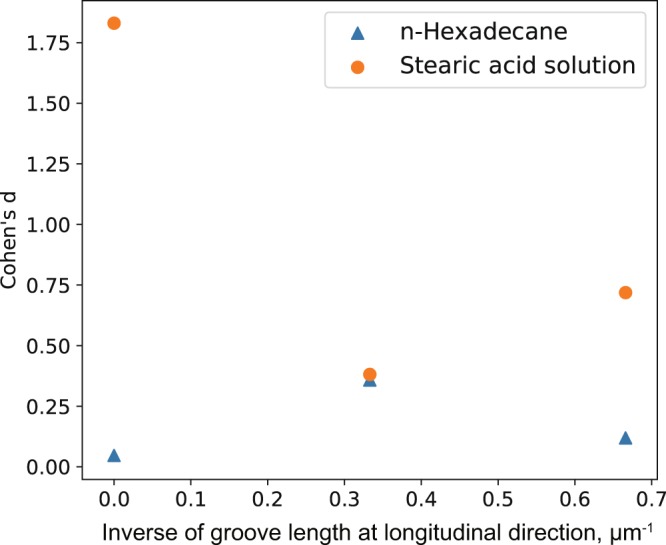
The relation of the inverse of groove length at longitudinal direction and Cohen’s *d* value.

As one of other factors of pattern shape, the difference in the groove length at the longitudinal direction could be thought. Focusing on the longitudinal ridge region, in case of the L&S pattern the groove length is infinity and there is no baffle which hinders fluid flow along grooves, on the other hands in cases of the L&S + B1 or L&S + B2 patterns the fluid could be dammed by the baffles. In the transverse ridge region, the groove length along a sliding direction is 500 nm for all patterns. To verify the influence of groove length at longitudinal direction on the lateral deflection, the relation between the Cohen’s *d* values and the inverse of groove length at the longitudinal direction for three patterns was plotted as Fig. 7. The inverse of groove length in the longitudinal region of L&S pattern was calculated as 1/∞ = 0 μm^−1^ and in the case of L&S + B2, the inverse was calculated as 1/1.5 μm ~ 0.67 μm^−1^. Since L&S + B1 pattern has half as many baffles as L&S + B2 pattern, the inverse of groove length was calculated as an average of 0 μm^−1^ and 0.67 μm^−1^ = 0.33 μm^−1^. The smaller inverse value means the fluid flows out easily along the grooves and the larger inverse value means the fluid in a groove is well hindered to flow out. From Fig. 7, in the case of the stearic acid solution, Cohen’s *d* value shows significant large value (1.83) at the inverse value is zero. At the larger inverse value, Cohen’s *d* value was so small that the gap of the frictional force between the longitudinal and transverse region is not significant. Therefore, at the situation that the fluid flows out easily along the grooves with no damming, the very large lateral deflection could be observed in stearic acid solution. On the contrary, once the fluid is dammed by the pattern ridges, the significant large value of lateral deflection was suppressed. From these results, the fluidity of fluid around pattern ridge could affect the frictional force in a stearic acid solution and the more fluid is dammed by ridge the less frictional force could be observed.

On the other hand, in the *n*-hexadecane environment, obvious difference in frictional force due to ridge direction and presence of baffles was not observed on all patterns including the L&S pattern. Therefore, the damming by a ridge of patterns has no influence on the frictional force in a *n*-hexadecane environment. The largest difference between a stearic acid solution and an additive-free *n*-hexadecane environment is that adsorbed molecular film is formed or not. Considering these aspects, the fluidity around pattern ridge could not effect on frictional force explicitly but on friction reducing effects of adsorbed molecular films, i.e., the more fluid is dammed by a ridge of patterns, the more friction reducing effect of adsorbed molecular film could be exerted. Based on this consideration, the reason why the transverse ridge region of the L&S pattern exhibited lower friction than the longitudinal one in stearic acid solution could be interpreted as follows; in the transverse ridge region, the fluid was dammed by the pattern ridges and this damming caused the more friction-reducing performance of the stearic acid than longitudinal ridge region where fluid was easily flowed out. However, the mechanism, why the damming effect effects on the friction-reducing performance of stearic acid remains as a question and further investigation is required.

## Supplementary information

Supplementary Infomation
