# Supplementary material for: In-Liquid Lateral Force Microscopy of Micropatterned Surfaces in a Fatty Acid Solution under Boundary Lubrication
Source: Sci Rep. 2019 Oct 23;9:15236. doi: 10.1038/s41598-019-51687-8 (PMC6811562; doi:10.1038/s41598-019-51687-8)
Supplement: Supplementary file 1 — Supplementary Infomation [file 41598_2019_51687_MOESM1_ESM.docx]

Supplementary information

In-Liquid Lateral Force Microscopy of Micropatterned Surfaces in a Fatty Acid Solution under Boundary Lubrication

Masaki Tsuchiko, Saiko Aoki

Department of Chemical Science and Engineering,

School of Materials and Chemical Technology, Tokyo Institute of Technology

S1-31, 12-1 O-okayama 2-chome, Meguro-ku, Tokyo 152-8552, Japan

*Corresponding Author: Saiko Aoki, saoki@chemeng.titech.ac.jp

Supplementary figures and table


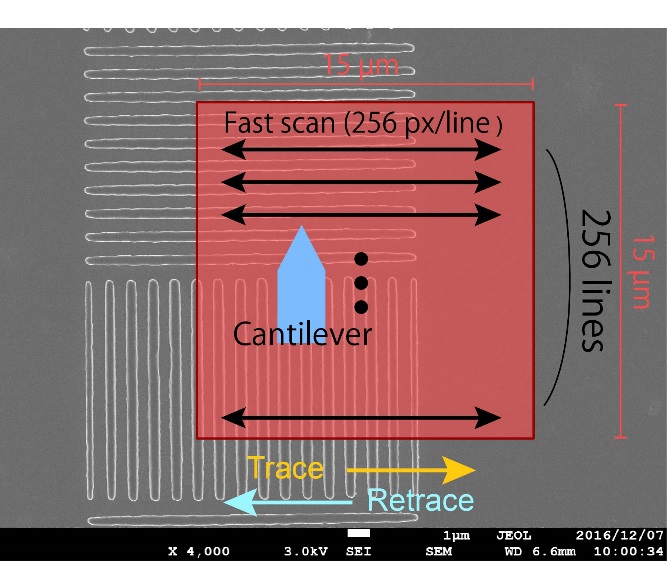


**Supplementary Fig. S1** Lateral force microscopy measurement area and scan direction.


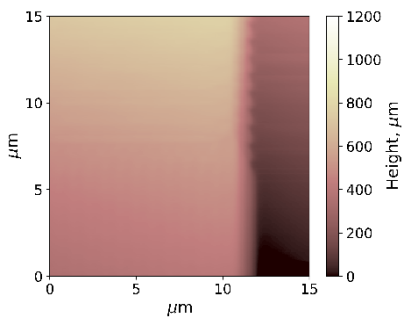


**Supplementary Fig. S2** Topographical image measured in *n*-hexadecane with a 63.8 μN normal load.


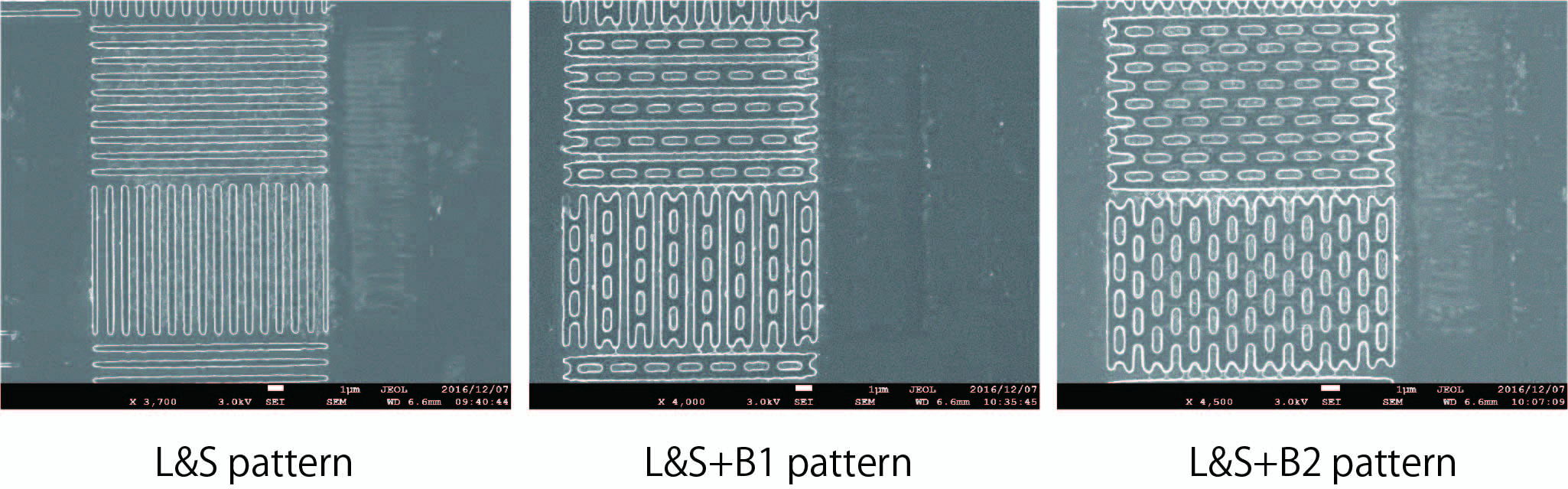


**Supplementary Fig. S3** SEM images of patterns after LFM measurements

**Supplementary Table S1** Lateral force microscopy (LFM) measurement conditions.

| Measurement area of LFM | 15 μm × 15 μm |
| --- | --- |
| Resolution | 256 × 256 pixels |
| Scan rate | 2 Hz (60 μm/s) |
| Normal load  (in *n*-hexadecane) | 12.8, 38.3, and 63.8 μN |
| Normal load  (in stearic acid solution) | 9.56, 28.7, and 67.0 μN |

Contact area calculation and maps

Supplementary Figs. S4 – S6 show color maps of contact area and models for calculation. The calculating area was set as 4000 nm ☓ 4000 nm and divided to 400 ☓ 400 cells (one cell is 10 nm ☓ 10 nm). The patterns and the plateau tip was modeled based on their dimension. The pattern and the plateau tip were overlaid, then the contact area was calculated as the overlap area. For example, Supplementary Fig. S4 lower figure shows contact model of the L&S pattern and the triangle plateau area, dark grey area representing overlap area (= contact area). To make contact area map, the triangle of the plateau tip was shifted to both X and Y direction and the contact area was calculated at each point, and then the calculated contact areas were plotted at the center of gravity of plateau tip triangle (Point G in Figs. S4 –S6) as color map (upper figure). The periodic boundary condition was applied for both X and Y axis.


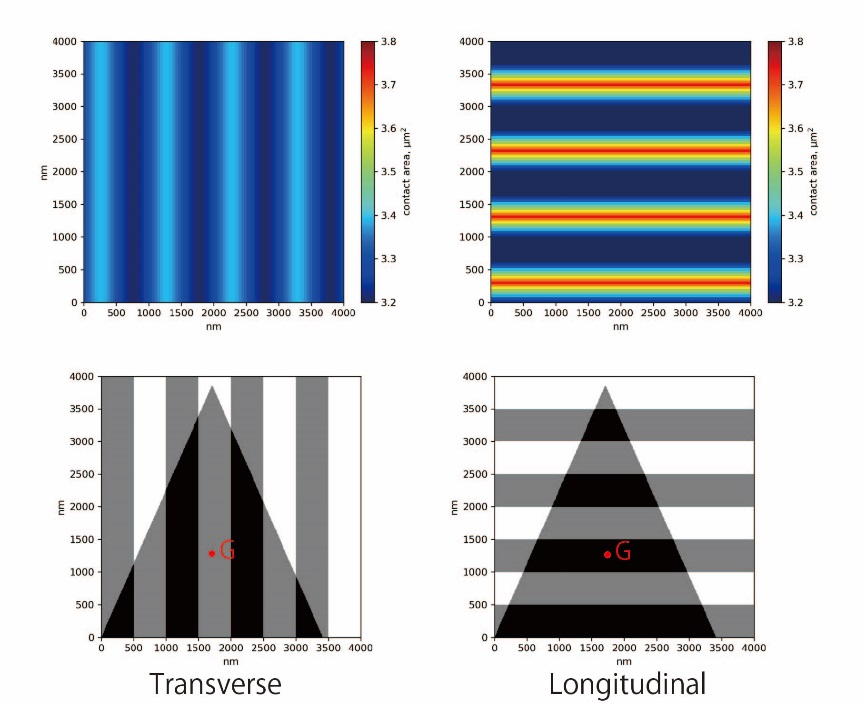


**Supplementary Fig. S4** Maps of contact area between L&S pattern and the plateau triangle(upper) and the model for calculation of area (lower)


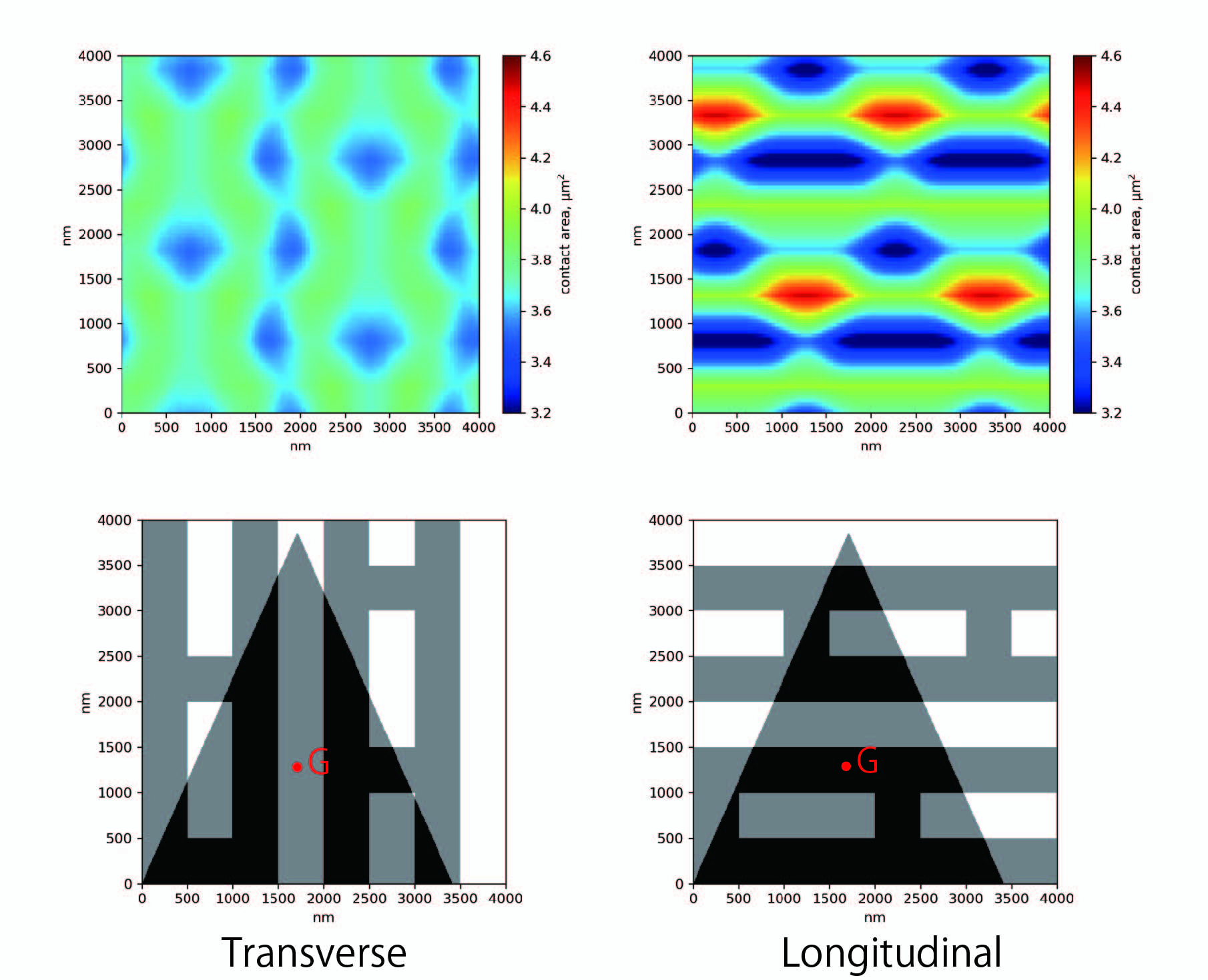


**Supplementary Fig. S5** Maps of contact area between L&S+B1 pattern and the plateau triangle(upper) and the model for calculation of area (lower)


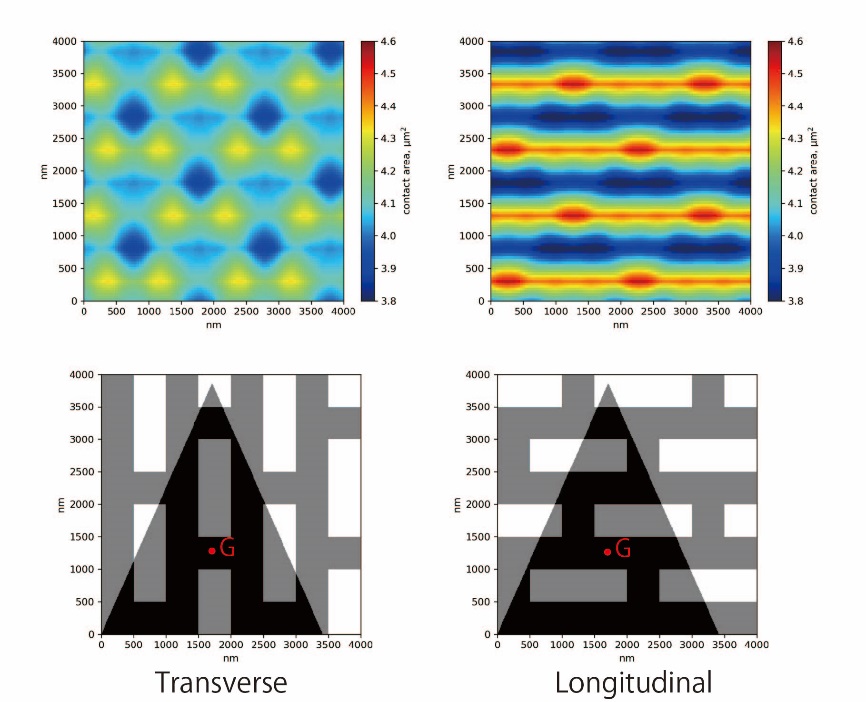


**Supplementary Fig. S6** Maps of contact area between L&S+B2 pattern and the plateau triangle(upper) and the model for calculation of area (lower)
